# Multiple Bony Mallet Finger Injuries in One Hand of a 14-Year-Old Boy

**DOI:** 10.7759/cureus.44441

**Published:** 2023-08-31

**Authors:** Jarallah M Albahlal, Abdullah Alzahrani, Hayat Khan

**Affiliations:** 1 Orthopedic Surgery, Dr. Sulaiman Alhabib Medical Group, Riyadh, SAU

**Keywords:** splint, k-wire, extension, multiple, mallet finger

## Abstract

Bony mallet finger injuries, commonly seen as isolated incidents, typically occur in active individuals. We report a rare case of simultaneous avulsion fractures at the distal phalangeal bases of the second, third, and fourth fingers on the right hand of a 14-year-old boy following a forced passive flexion injury during a football game. The patient initially received conservative management with a finger extension splint for the distal interphalangeal (DIP) joints. However, one week after the injury, we performed surgical fixation on all affected digits using the K-wire extension block method due to multiple fractures and the patient's intolerance for the mallet finger splint. After six weeks, all K-wires were removed, and physiotherapy sessions began. Three months post-injury, the second and fourth DIP joints demonstrated an “Excellent" outcome, and the third DIP joint demonstrated a "Good" outcome based on Crawford’s criteria for outcome assessment of mallet finger injury after management. This case highlights the importance of early detection and appropriate management of concomitant mallet finger injuries in pediatric patients to prevent potential complications that could impair hand function and quality of life.

## Introduction

Avulsion fractures at the base of the distal phalanx, also known as bony mallet finger injuries, are prevalent hand injuries, particularly among active individuals [1,2]. Rarer still are multiple avulsion injuries affecting fingers of the same hand, which clinicians often overlook due to their infrequent occurrence [3]. We report a case of a 14-year-old boy who experienced multiple avulsion fractures on the second, third, and fourth distal phalangeal bases of his right hand following a forced passive flexion injury and their successful management by surgical K-wire extension block fixation.

## Case presentation

**Figure 1:**
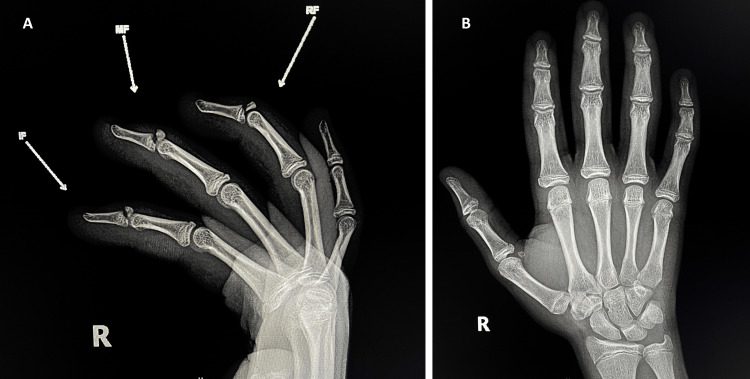
Right hand x-ray of a 14-year-old boy showing multiple bony mallet finger injuries at the base of the second, third, and fourth distal phalanges (white arrows). (A) Lateral view, (B) Anteroposterior view. Abbreviations: IF, index finger; MF, middle finger; RF, ring finger.

**Figure 2:**
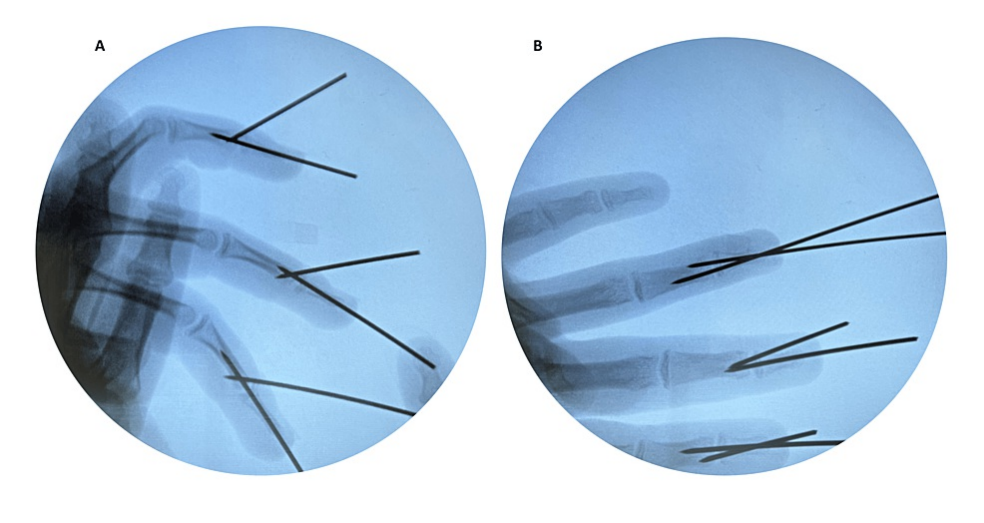
Intraoperative x-ray showing fixation of the second, third, and fourth distal phalanges using the K-wire extension block technique. (A) Lateral view, (B) Anteroposterior view

**Figure 3:**
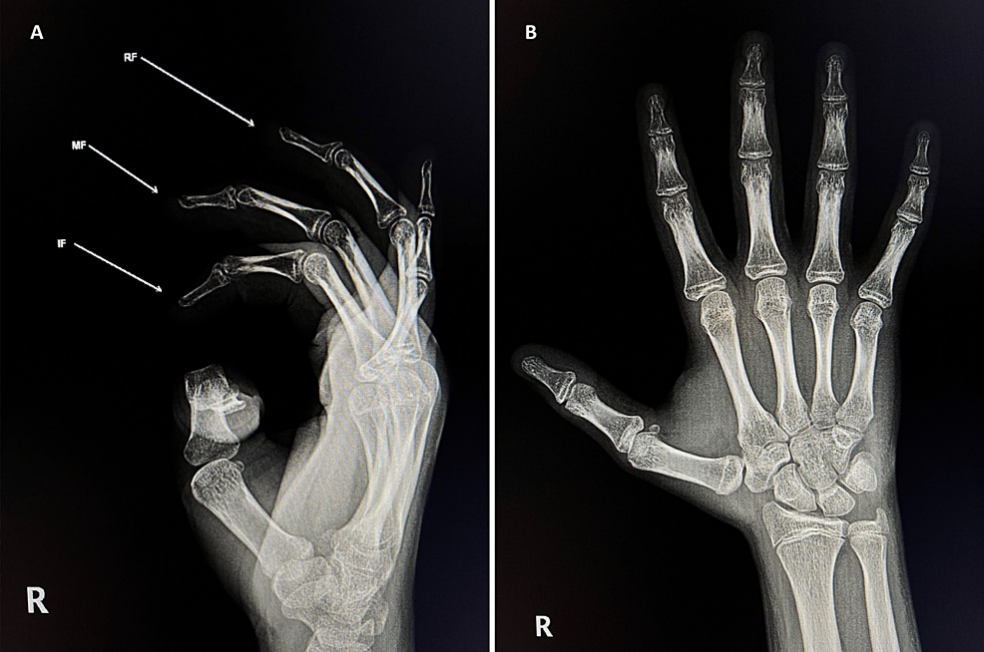
Three-month follow-up x-ray. (A) Lateral view, (B) Anteroposterior view. Abbreviations: IF, index finger; MF, middle finger; RF, ring finger.

**Figure 4:**
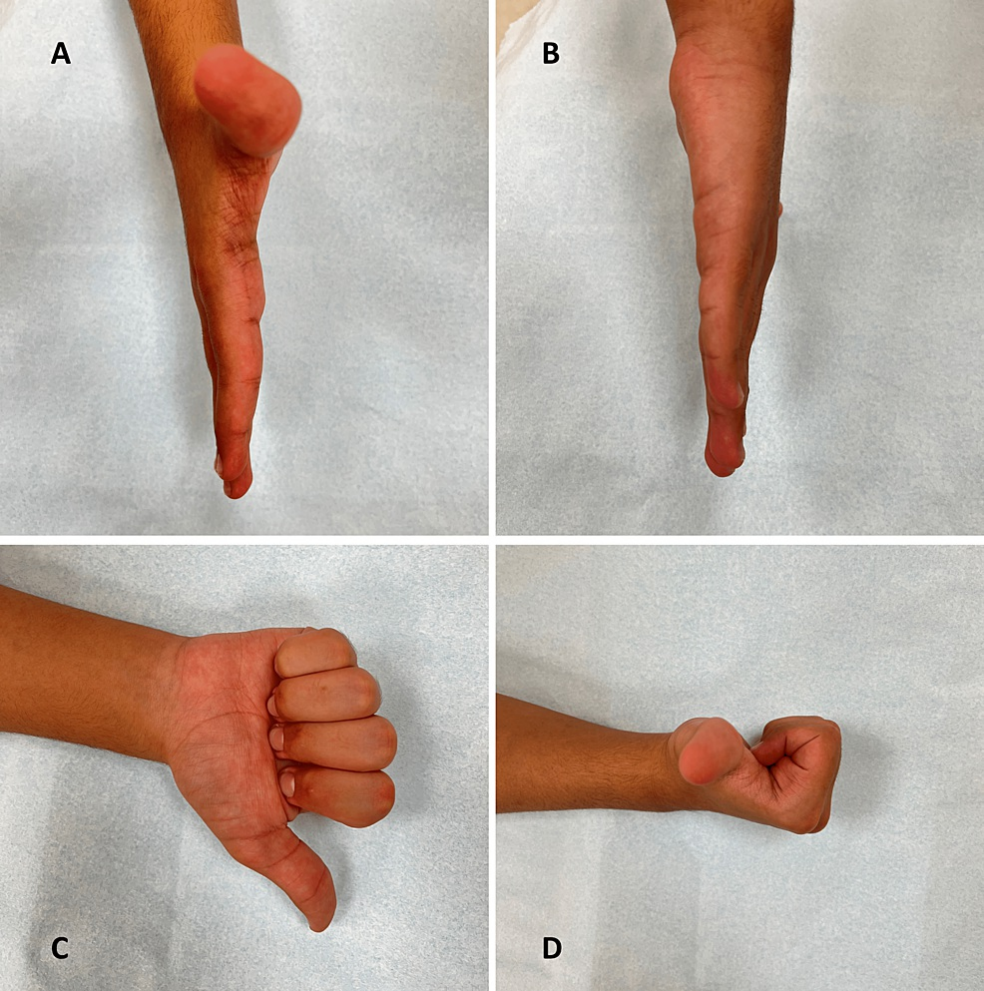
Clinical picture of the affected hand. (A) Lateral view, (B) Medial view, (C) Anterior view in full flexion of fingers, (D) Lateral view in full flexion of fingers.

A 14-year-old boy without any previous medical or surgical history presented to the emergency department with concerns of pain and limited mobility in his right dominant hand's second, third, and fourth distal phalanges. The injury occurred during a football game when he sustained a forced passive flexion. Physical examination revealed tenderness, coupled with pain-induced immobility in the affected fingers. No wounds or open fractures were evident. X-ray images revealed avulsion fractures at the dorsal base of the distal phalanx of the second, third, and fourth fingers (Figure 1). Based on the Wehbe and Schneider classification of mallet finger, all the injured fingers have a type 1B fracture (Table 1) [4]. The patient received treatment initially with a finger extension splint for the distal interphalangeal (DIP) joints. One week after the injury, we performed surgical fixation on all affected digits using the K-wire extension block method (Figure 2). We applied a short arm splint on the volar side. We removed all K-wires in the clinic six weeks post-fixation and initiated range of motion and strengthening physiotherapy sessions for the affected hand. Three months later, an x-ray confirmed bone healing (Figure 3). The third DIP joint exhibited a 10-degree extension lag, full flexion, and no pain, qualifying as a “Good” outcome based on Crawford’s criteria (Figure 4, Table 2). However, the second and fourth DIP joints met the “Excellent” outcome rating based on Crawford's criteria, which stipulate a full extension, full flexion, and an absence of pain [5].

**Table 1:** Wehbe and Schneider classification for mallet finger injury. Abbreviation: DIP, distal interphalangeal. [4]

Type/Subtype	Description
I	No DIP joint subluxation
II	DIP joint subluxation
III	Epiphyseal & physeal injuries
A	< 1/3 of articular surface involvement
B	1/3 to 2/3 of articular surface involvement
C	> 2/3 of articular surface involvement

**Table 2:** Crawford’s criteria for outcome assessment of mallet finger injury after management. [5]

Grade	Outcome measure		
	Pain	Extension deficit	Flexion deficit
Excellent	No pain	0°	0°
Good	No pain	0°-10°	0°
Fair	No pain	10°-25°	> 0°
Poor	Persistent pain	>25°	≥0°

## Discussion

"Mallet" finger injuries, so named for resembling a hammer, commonly occurred in sports-related incidents in the 1800s [6]. These injuries can be either ligamentous or bony avulsions resulting from disruption of the terminal extensor mechanism at the base of the distal phalanx [7]. Multiple mallet finger injuries can be missed if not detected, and unrecognized mallet finger injuries can affect grip strength and potentially lead to early DIP joint osteoarthritis and ultimately swan neck deformity, impairing hand function and overall quality of life.

Conservative management for soft tissue mallet finger injuries typically involves six to eight weeks of splinting. Bony mallet finger injuries, on the other hand, can be treated either conservatively with splinting or through surgical fixation, with neither approach showing significant differences in pain, DIP joint extensor lag, or range of motion [8]. However, surgical fixation is favored in cases of open or otherwise complex mallet finger injuries, such as displaced bone fragment, risk of displacement of bone fragment over one-third of the distal phalanx articular surface, and volar subluxation of the distal phalanx [9]. In this case, we opted for surgical fixation due to the patient's multiple mallet finger fractures and intolerance for the mallet finger splint; given that his injuries involved multiple fingers in his dominant hand, he removed the splint multiple times during the splinting period, prolonging the immobilization period and increasing the likelihood of DIP joint stiffness.

There are multiple surgical fixation techniques for bony mallet finger, but the most commonly used methods are closed reduction with K-wire extension block fixation and open reduction with direct pinning fixation. Both techniques achieve an equal anatomical reduction in acute mallet finger cases (less than three weeks old). Open reduction and direct pinning are time-consuming and may lead to bone fragment comminution during fixation, potentially resulting in loss of fixation. However, this approach can provide an earlier DIP joint range of motion and a lower infection rate than closed reduction and K-wire extension block fixation [10]. In this case, we applied the K-wire extension block technique as described by Ishiguro et al. [11] but made a minor modification. Instead of using the ulnar or radial entry point described by Ishiguro et al., we chose a more distal and straight entry point for the distal phalanx K-wire to immobilize the DIP joint. This modification was made to prevent the irritation of adjacent soft tissue, especially since we treated multiple consecutive fingers. The adapted extension block technique effectively and quickly reduced and fixed all the digits affected by bony mallet finger injury.

## Conclusions

Bony mallet finger injuries typically affect a single digit. The simultaneous affliction of three digits on one hand is rare. In our study, the patient was a 14-year-old boy who suffered second, third, and fourth bony mallet finger injuries on his right dominant hand. The injury was initially managed conservatively but was later converted to surgical fixation using the K-wire extension block technique. This case underscores the necessity of early detection by careful examination and appropriate management of concurrent mallet finger injuries, particularly in pediatric patients. Such injuries, though rare, could go unnoticed, particularly when affecting the dominant hand. Young age compounds the challenge of managing these cases, heightening the risk of complications that could impair hand function and quality of life. Hence, vigilance in identification and treatment is crucial.
